# Clonal Evolution of *TP53* c.375+1G>A Mutation in Pre- and Post- Neo-Adjuvant Chemotherapy (NACT) Tumor Samples in High-Grade Serous Ovarian Cancer (HGSOC)

**DOI:** 10.3390/cells8101186

**Published:** 2019-10-01

**Authors:** Marica Garziera, Erika Cecchin, Giorgio Giorda, Roberto Sorio, Simona Scalone, Elena De Mattia, Rossana Roncato, Sara Gagno, Elena Poletto, Loredana Romanato, Fabrizio Ecca, Vincenzo Canzonieri, Giuseppe Toffoli

**Affiliations:** 1 Experimental and Clinical Pharmacology Unit, Centro di Riferimento Oncologico (CRO), IRCCS, 33081 Aviano, Italy; ececchin@cro.it (E.C.); edemattia@cro.it (E.D.M.); rroncato@cro.it (R.R.); sgagno@cro.it (S.G.); lromanato@cro.it (L.R.); fabrizio.ecca@cro.it (F.E.); gtoffoli@cro.it (G.T.); 2 Gynecological Oncology Unit, Centro di Riferimento Oncologico (CRO), IRCCS, 33081 Aviano, Italy; ggiorda@cro.it; 3 Medical Oncology Unit C, Centro di Riferimento Oncologico (CRO), IRCCS, 33081 Aviano, Italy; rsorio@cro.it (R.S.); sscalone@cro.it (S.S.); 4 Medical Oncology, “Santa Maria della Misericordia” University Hospital, ASUIUD, 33100 Udine, Italy; polettoelena@libero.it; 5 Pathology Unit, Centro di Riferimento Oncologico (CRO), IRCCS, 33081 Aviano, Italy; vcanzonieri@cro.it; 6 Department of Medical, Surgical and Health Sciences, University of Trieste, 34127 Trieste, Italy

**Keywords:** HGSOC, NACT, NGS, *TP53*, chemoresistance

## Abstract

Carboplatin/paclitaxel is the reference regimen in the treatment of advanced high-grade serous ovarian cancer (HGSOC) in neo-adjuvant chemotherapy (NACT) before interval debulking surgery (IDS). To identify new genetic markers of platinum-resistance, next-generation sequencing (NGS) analysis of 26 cancer-genes was performed on paired matched pre- and post-NACT tumor and blood samples in a patient with stage IV HGSOC treated with NACT-IDS, showing platinum-refractory/resistance and poor prognosis. Only the *TP53* c.375+1G>A somatic mutation was identified in both tumor samples. This variant, associated with aberrant splicing, was in *trans* configuration with the 72Arg allele of the known germline polymorphism *TP53* c.215C>G (p. Pro72Arg). In the post-NACT tumor sample we observed the complete expansion of the *TP53* c.375+1G>A driver mutant clone with somatic loss of the treatment-sensitive 72Arg allele. NGS results were confirmed with Sanger method and immunostaining for p53, BRCA1, p16, WT1, and Ki-67 markers were evaluated. This study showed that (i) the splice mutation in *TP53* was present as an early driver mutation at diagnosis; (ii) the mutational profile was shared in pre- and post-NACT tumor samples; (iii) the complete expansion of a single dominant mutant clone through loss of heterozygosity (LOH) had occurred, suggesting a possible mechanism of platinum-resistance in HGSOC under the pressure of NACT.

## 1. Introduction

Epithelial ovarian cancer is the most lethal malignancy of the female genital tract and the eighth leading cause of cancer-related death in 2018, among women worldwide [1]. High-grade serous ovarian cancer (HGSOC) accounts for up to 60–70% of ovarian carcinomas [2]. Most patients receive a diagnosis at an advanced stage (III–IV) of the disease because of a lack of specific symptoms, ending in a poor prognosis with a five-year survival rate of only 45% [3]. Despite an initial good response to platinum-based chemotherapy relapse seems unavoidable, with ~70% of patients experiencing disease recurrence in the first two years since diagnosis and requiring further treatments [4]. Primary debulking surgery (PDS), followed by platinum-based chemotherapy, is the standard treatment for advanced ovarian cancer, including HGSOC. Residual disease (RD) is a major prognostic factor for survival with no visible tumor residue (RD = 0) as the goal of surgery [5]; nevertheless, a complete resection is very difficult to achieve in patients with massively disseminated tumors [6]. Neo-adjuvant chemotherapy (NACT) based on a platinum/taxane combination, prior to interval debulking surgery (IDS), is an alternative treatment option in patients with stage III–IV advanced ovarian cancer who are not candidates for PDS, due to unresectable disease and/or poor performance status [7,8]. The benefits from NACT-IDS, compared to PDS followed by standard chemotherapy, are still controversial. Although two randomized trials [9,10] found an equivalent overall survival (OS) and reduced surgical morbidity and one study showed mortality reduction with NACT [11], in several other studies [8,12,13,14], less favorable outcomes, such as decreased OS, and an increased toxicity due to NACT, were reported when comparing the two treatment options. Furthermore, some studies have shown an increased risk of developing platinum resistance in stage III–IV ovarian cancer after NACT [6,8], and the in vitro induction of platinum and paclitaxel resistance after NACT and IDS, respectively [15,16]. The development of chemotherapy-refractory/resistant clones is a critical barrier to cure patients with advanced ovarian cancer, in particular HGSOC. However, relatively little is known of the genomic evolution of HGSOC under the selective pressure of chemotherapy. Although somatic mutations in *TP53* are a frequent event in these patients [17,18] and ~20% are also mutated in the *BRCA1/2* susceptibility genes, due to a combination of germline and somatic mutations [19], extensive intratumoral heterogeneity in primary HGSOCs has been documented by the use of high-throughput sequencing techniques [20,21,22,23]. Next-generation sequencing (NGS) technology has expanded the knowledge of the complex genomic heterogeneity of ovarian cancer through the discovery of candidate targets for future therapeutic applications. In addition, the identification of germline and somatic mutations better define the subtype-specific molecular signatures, highlighting mechanisms of treatment resistance influencing ovarian and gynecologic malignancies [24]. This study, using targeted NGS, analyzed matched pre- and post-NACT ovarian tumor specimens and matched blood samples, from a patient with HGSOC characterized by a poor response to treatment and early death, within a 26 cancer-genes panel [23,25]. The aim was to compare tumor molecular profiles before and after NACT, based on the carboplatin/paclitaxel combination, in order to evaluate its influence in determining the platinum-refractory/resistance and outcomes in the HGSOC. A somatic mutation and a germline polymorphic variant coupled in *trans* configuration (i.e., located in different chromosomes) were identified in *TP53* and also confirmed by standard Sanger sequencing. Through loss of heterozygosity (LOH, i.e., allelic imbalance or copy-neutral LOH), we observed the expansion of the founder clone with the *TP53* driver mutation which was coupled with the loss of the treatment-sensitive variant germline allele, suggesting a mechanism of chemoresistance in HGSOC. Moreover, to better characterize the HGSOC, immunostaining for p53 (in pre- and post-NACT tumor samples) and for BRCA1, p16, Wilms tumor 1 (WT1) and Ki-67 markers in chemo-naïve tumor, were also determined. Patients with *trans* configuration of the *TP53* variants identified by NGS in the tumor are at high risk of mutation selection by NACT based on platinum/paclitaxel combination therapy. This study may have relevant impact in translational medicine and reveal a clinical applicability in the choice of treatment in HGSOC and advanced ovarian cancer.

## 2. Materials and Methods

### 2.1. Sample Collection and Human Ethics

The patient included in this report was diagnosed and treated at CRO Institute between February 2005 and August 2007. Tumor and blood matched samples were collected at diagnostic laparoscopy (D-LPS) and IDS and retrospectively analyzed. Tumor staging and tumor grading were assessed according to Fédération Internationale de Ginécologie et d’Obstetrique (FIGO) and to WHO (World Health Organization) criteria respectively. Clinico-pathological characteristics, treatment, and complete follow-up information were collected from the medical record as current clinical surveillance procedures. OS was defined as the interval between diagnosis (at D-LPS) and the date of death. Time to recurrence (TTR) was defined as the interval between IDS and the date of the first recurrence/progression. Platinum-free interval (PFI) was defined as the interval between the end of the first-line platinum-based treatment after IDS and the date of first recurrence/progression. The patient was defined as “platinum-resistant” because relapse occurred within < 6 months from the end of platinum treatment [23]. Clinical genetic testing of germline mutations in *BRCA1/2* genes was not performed at the time of the enrollment because the patient had no family history of breast and/or ovarian cancer [23]. Written informed consent was obtained from the patient with histologically confirmed epithelial ovarian cancer for the use of peripheral blood, tissue samples, and clinical data for research purposes. The study was conducted in accordance with the Declaration of Helsinki and was approved by the Ethics Committee of the CRO Aviano National Cancer Institute, Italy (Institutional Review Board n. CRO-2014-43).

### 2.2. Next Generation Sequencing Analysis and Somatic LOH Analysis

Single nucleotide variants (SNVs) in *TP53* for this patient with HGSOC emerged during a genomics screen aimed to identify new genetic markers of platinum resistance and patient prognosis in epithelial ovarian cancer using a targeted NGS approach [23]. The patient was not included in the previous study [23] being treated with NACT-IDS. The Illumina TruSight Tumor 26-genes panel (Illumina, Inc., San Diego, CA, USA; http://www.illumina.com/products/trusight-tumor-26-gene.html) was chosen for NGS analysis, as recently reported [23]. This panel provides coverage of exon coding regions, where variation has been cataloged in the COSMIC database in oncogenes, and coverage of all 11 exons and intronic flanking regions of *TP53* tumor suppressor gene. TruSight Tumor 26-genes panel gives a more comprehensive view of somatic variation in solid tumors, including lung, colon, melanoma, gastric, and ovarian cancer.

Frozen ovarian tumor specimens taken at D-LPS (pre-NACT) and during IDS (post-NACT) and a matched blood sample were analyzed retrospectively. As a WT reference sample, genomic DNA was isolated from mononuclear cells of matched peripheral blood samples collected at the time of primary surgery. Tumor samples were macrodissected and visually inspected by the pathologist to assess a minimum tumor cellularity of 70%. Genomic DNA was extracted from both the tumor and blood using the EZ1 DNA Tissue Kit and EZ1 DNA Blood 350 µL Kit (Qiagen, Hilden, Germany), respectively, according to the manufacturer’s instructions. DNA samples were quantified using PicoGreen Dye (Quant-iT PicoGreen dsDNA Assay Kit, Thermo Fisher Scientific, Waltham, MA, USA) on a Tecan Infinite 200 PRO reader (Tecan Trading AG, Männedorf, Switzerland) and normalized to 5 ng/μL for successive library preparation. DNA libraries were prepared for NGS according to the manufacturer’s instructions. This panel (21 Kb size) screens 82 exons (all 11 exons of *TP53* and intronic boundaries) in 26 tumor-related genes (*AKT, ALK, APC, BRAF, CDH1, CTNNB1, EGFR, ERBB2, FBXW7, FGFR2, FOXL2, GNAQ, GNAS, KIT, KRAS, MAP2k1, MET, MSH6, NRAS, PDGFRA, PIK3CA, PTEN, SMAD4, SRC, STK11, TP53*) across 174 amplicons (165–195 bp in size) with a 1000x minimum coverage (mean 7000x) of each amplicon. Normalized libraries were analyzed on a MiSeq platform (Illumina) using a V3 (600 cycles) sequencing flow cell with a 2 × 121 base pairs analysis set-up. The raw data were automatically processed and analyzed by the Illumina-Miseq system pipeline. VCF files were imported in Variant Studio software (Illumina Variant Studio Data Analysis Software 2.2) for variant calling and imputation. The filtering of genetic variants was performed per the following criteria: PASS filter, variant call quality equal to 100, frequency of the Alternative (Alt) allele greater or equal to 4% (TruSight Tumor panel achieves limits of detection below 5% variant allele frequency with a minimum cut-off point of 3%), and the total number of reads passing quality filters (read depth) greater or equal to 1000x. COSMIC (https://www.cancer.sanger.ac.uk/cosmic), dbSNP (https://www.ncbi.nlm.nih.gov/snp, ClinVar databases (http://www.ncbi.nlm.nih.gov/clinvar/) and IARC TP53 Mutation Database (http://p53.iarc.fr/ProtocolsandTools.aspx), were searched to determine whether the detected variants were previously assigned ID numbers. Somatic LOH was determined comparing the same heterozygous and informative SNVs detected in germline vs tumor samples: an allele was considered imbalanced or relatively lost when significant changes in individual variant allele frequency (VAF), defined as absolute increases or decreases of at least 15 in the tumor, respectively, were observed as previously described [26]. We checked for statistically significant differences between pre- and post-NACT using Fisher’s exact test on a 2 × 2 table of allele type (reference and variant) vs sample type (tumor and blood) as previously reported [27].

### 2.3. Sanger Sequencing to Validate TP53 SNVs in Trans Configuration

Sanger sequencing was performed to validate the double *TP53* SNVs identified in *trans* configuration using genomic DNA extracted from patient’s tumor tissues and blood. Variants were confirmed by at least two independent PCR amplifications and a DNA sequencing reaction on both strands. Primers used to amplify *TP53* exon 4 were downloaded from the public “IARC TP53 Database” (http://p53.iarc.fr/ProtocolsandTools.aspx), TP53ex4F: 5′-TGAGGACCTGGTCCTCTGAC-3′ and TP53ex4R: 5′-AGAGGAATCCCAAAGTTCCA-3′. Target region was PCR amplified from extracted genomic DNA; each 50 µL reaction contained 1.8 mM MgCl_2_, 0.25 mM of each dNTP, 0.5 µM of each primer, 20–200 ng of genomic purified DNA template, 1 × PCR Buffer, 0.2 µL of AmpliTaq Gold DNA polymerase (Applied Biosystems, Foster City, CA, USA), and MilliQ water. The PCR protocol was as follows: initial denaturation at 94 °C for 2 min, then 50 cycles of subsequent denaturation at 95 °C for 30 s, annealing at 57 °C for 45 s, and extension at 72 °C for 1 min, followed by the final extension step at 72 °C for 10 min. The PCR products (5 µL) were electrophoresed in 3% agarose and amplified samples cleaned up using ExoProStar (GE Healthcare). Purified reactions (1–2 μL) were sequenced using the Big Dye Terminator kit (Applied Biosystems, Foster City, CA, USA) and an ABI PRISM capillary sequencer. Sequencing chromatograms were visualized with Chromas software version 2.01 (Technelysium Pty Ltd., South Brisbane, Australia) and aligned with the human *TP53* reference genomic sequence.

### 2.4. Immunohistochemistry

One tumor-rich sample per case (pre-NACT at D-LPS and post-NACT at IDS), a 4-µm-thick section from a formalin-fixed or bouin-fixed, paraffin-embedded tumor tissue block, were selected for p53 immunohistochemical analysis. One tumor-rich sample collected before therapy (pre-NACT at D-LPS) was used for BRCA1, WT-1, p16 and Ki-67 immunohistochemical analysis, respectively. Immunoperoxidase labeling was performed with the automated XT iVIEW DAB V.1 procedure on the BenchMark ULTRA IHC/ISH Staining Module, Ventana with anti-p53 (clone Bp53-11, prediluted, Ventana, Innovation Park Dr. Tucson, AZ, USA), for pre- and post-NACT tumor samples, and with anti-BRCA1 (clone MS110 prediluted, Abcam, Discovery Drive, CB, UK), anti-p16 (clone E6H4, ready to use, Ventana), anti-Wt1 (clone 6F-H2 ready to use, Ventana), anti-Ki-67 (clone 30-9 ready to use, Ventana), for only the pre-NACT tumor sample. Sections were incubated with: anti-p53 for 16 min at 37 °C, anti-BRCA1 for 36 min at room temperature, anti-p16 for 16 min at 36 °C, anti-WT1 for 40 min at 37 °C, and anti-Ki-67 for 32 min at 42 °C. Antigen retrieval was carried out with CC1 (Ventana) for all primary antibodies. Stainings were detected using the I-View DAB detection system. All slides were reviewed by the pathologist (VC), who was blinded to molecular data. Nuclear staining was considered a positive reaction. The extent of nuclear or cytoplasmic staining was estimated to the nearest 5% level of positive tumor cells, reporting the actual percentage for each case. The intensity of staining was recorded as - (Absent); -/+ (Weak); + (Moderate); ++ (High); or +++ (Very High).

## 3. Results

### 3.1. Case History

**Figure 1:**
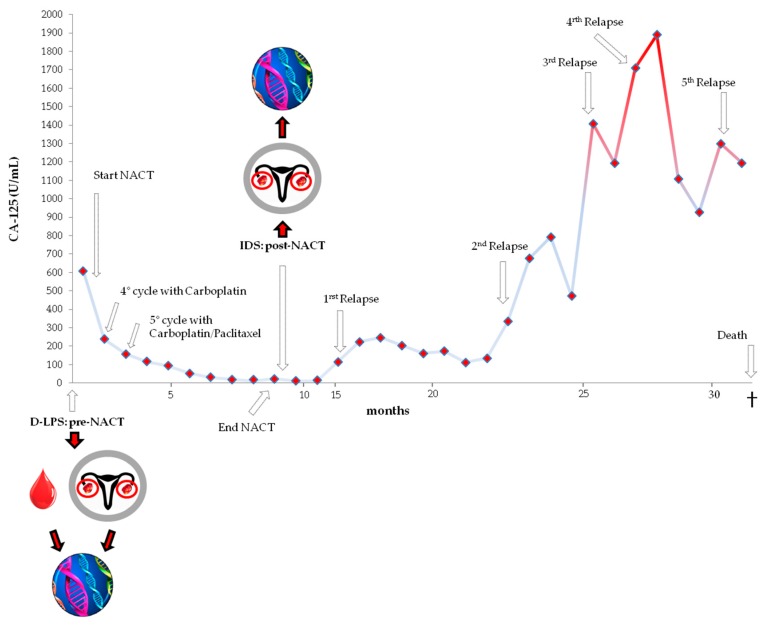
Graphical representation of CA-125 levels across the disease/treatment course of the patient diagnosed with HGSOC. The two time points, at D-LPS (pre-NACT) and at IDS (post-NACT), in which blood and ovarian tumor tissue samples were collected, are highlighted. CA-125: cancer antigen 125; D-LPS: diagnostic laparoscopy; NACT: neo-adjuvant chemotherapy; IDS: interval debulking surgery; HGSOC: high-grade serous ovarian cancer.

A 63-year-old patient was referred to CRO Aviano-National Cancer Institute for suspected advanced ovarian carcinoma with presence of abundant abdominal and pleural ascites (clinical stage IV). She denied familial history of ovarian and/or breast cancer. In February 2005, through an explorative D-LPS, the patient was deemed unresectable because of diffuse carcinomatosis with deeply infiltrating nodules, thus, undergoing only aspiration of hemorrhagic ascites (~2300 mL), right ovariectomy, partial omentectomy, and the collection of multiple biopsies. Then, NACT was recommended. The pathological diagnosis was poor differentiated (tumor grade G3) infiltrating ovarian adenocarcinoma (pT3cNx) of the serous sub-type, and the patient was assigned to FIGO stage IV for the presence of distal pleural effusion. CA-125 level (normal value is < 35 U/mL) before chemotherapy was 608.0 U/mL (Figure 1).

The patient underwent four cycles of NACT (carboplatin alone, area under curve (AUC) 5 with one intrapleural administration of Bleomycin before the second cycle of NACT), that was then prolonged with six additional cycles of carboplatin/paclitaxel (carboplatin AUC 5 and paclitaxel 255 mg/m^2^), due to poor clinical response to platinum based on Response Evaluation Criteria In Solid Tumors (RECIST) criteria of computed tomography (CT) scan and CA-125 level, still high (239.0 U/mL). After a total of ten cycles, the chemotherapeutic response was only partial with normalization of CA-125 level but with positron emission tomography (PET)/CT scan still revealing multiple peritoneal nodules and pleural effusion. According to the National Cancer Institute-Common Toxicity Criteria version 2.0 (NCI-CTC v. 2.0), the patient experienced grade G4 neutropenia after the 5th, 7th, and 9th cycle, G1 and G2 alopecia after the 5th and the 6th cycle respectively, and G1 fatigue after the 9th cycle. Neurotoxicity was not reported. Surgery (IDS) was then attempted with hysterectomy, left ovariectomy, appendectomy, and partial intestinal resection for presence of peritoneal and mesenteric nodules (RD < 0.5 cm). Upon the histological assessment of the surgical specimens, the ovarian cancer was classified as poor differentiated/undifferentiated (G3/4), FIGO stage IIIC (ypT3cNx) serous ovarian cancer. After IDS three additional cycles with only carboplatin were administered to the patient. Disease progression was detected in peritoneum with increased dimension of peritoneal nodules (revealed by PET/CT) and also evidenced by the biochemical increase in CA-125 marker (116.0 U/mL), just less than five months from the last carboplatin administration. The patient was defined as platinum-resistant (PFI 4.4 months) and TTR was 7.5 months. The patient received a second-line chemotherapy with carboplatin/PDL (Pegylated liposomal Doxorubicin) but after the eighth cycle the treatment was stopped for disease progression (peritoneal dissemination and CA-125 increase, 334.3 U/mL). The patient was then treated with Capecitabine but after three cycles the chemotherapy was interrupted for disease progression (peritoneal dissemination and CA-125 increase, 1407.0 U/mL). Then, hormonal therapy with Letrozole was given to the patient for about one month until the detection of progression (peritoneal dissemination and CA-125 increase, 1711.0 U/mL). Finally, the patient received three cycles of Gemcitabine which was stopped (disease progression) and substituted with palliative care until death. The OS was 30.7 months.

### 3.2. Molecular Analysis by NGS

NGS was performed on frozen ovarian cancer tissues of the patient collected at the D-LPS (pre-NACT) and during IDS (post-NACT). A blood sample collected at D-LPS was used as a matched control/reference. Analysis of the 26 tumor-related genes on the targeted NGS panel (BRCA1/2 were not included in the commercial panel) was carried out using Variant Studio after filtering genetic variants using the following criteria: PASS filter, variant call quality = 100, VAF of the Alt allele ≥ 4%, and total number of reads passing quality filters (read depth) ≥1000x. Among the 26 tumor-related genes, one somatic SNV was detected only in *TP53*. Another germline SNV was detected in tumor samples and in the matched normal/blood sample. VAF was calculated in each sample as the ratio of the number of sequence reads documenting the alternative allele versus all sequence reads aligned to that position. SNVs were automatically annotated against the human *TP53* reference genomic sequence NC_000017.10 (chr 17: 7,571,720-7,590,868) corresponding to isoform NM_000546.5 (Table 1).

**Figure 2:**
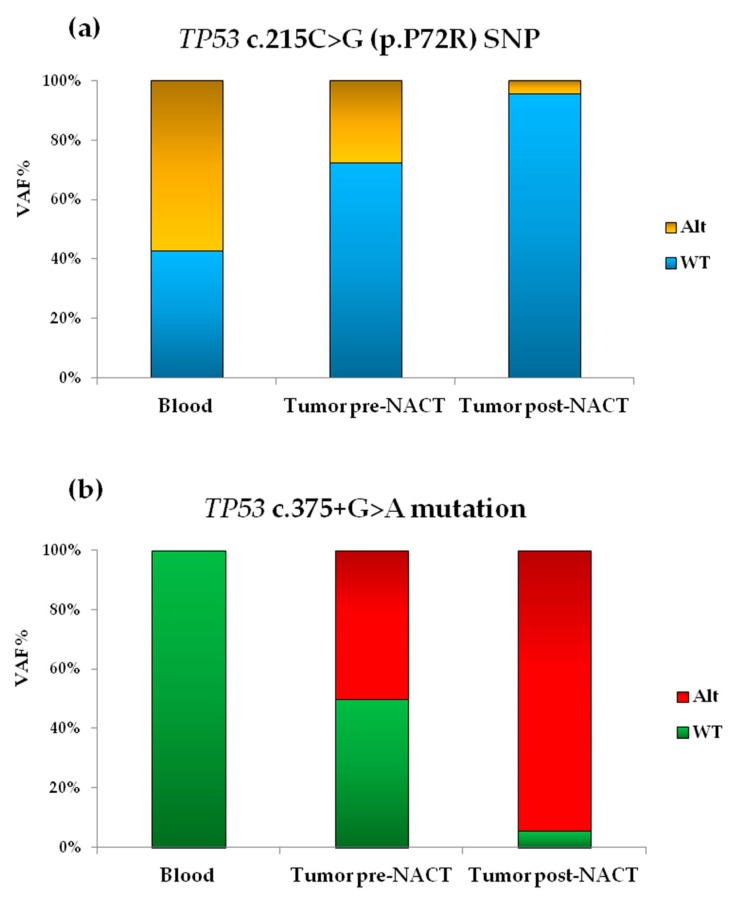
Graphic representation of VAF observed for SNVs in *TP53* identified by NGS in blood (reference) and tumor tissue samples, collected at pre-therapy (pre-NACT) at the D-LPS and post-therapy (post-NACT) at the IDS, from a patient with HGSOC. (**a**) Bar-graph shows VAF decrease in pre- (27.71%) and post-NACT (4.21%) tumor samples, indicating prevalence of the WT “*C*” allele (blue bar) compared to the Alt “*G*” allele (yellow bar) at IDS for the *TP53* c.215C>G (p.P72R) SNP, present in heterozygosis (57.31%) in the blood reference sample; (**b**) Bar-graph shows VAF increase in pre- (50.13%) and post-NACT (94.33%) tumor samples highlighting prevalence of the Alt “*A*” allele (red bar) compared to the WT “*G*” allele (green bar) in the HGSOC tissue sample collected at IDS of *TP53* c.375+1G>A mutation, absent in the blood reference sample (only WT “*G*” allele, green bar). VAF: variant allele frequency; NACT: neo-adjuvant chemotherapy; HGSOC: high-grade serous ovarian cancer; D-LPS: diagnostic laparoscopy; IDS: interval debulking surgery; Alt: alternative; WT: wild-type.

The somatic mutation *TP53* c.375+1G>A (i.e., *TP53* IVS4+1G>A) was detected in pre- and post-NACT tumor samples from HGSOC. This mutation affects the first nucleotide downstream exon 4 that belongs to a canonic splice donor (SD) site, consisting of two conserved GT nucleotides. This variant has been already reported both in the International Agency for Research on Cancer (IARC) TP53 Mutation Database (Database R19, released August 2018) [28] and in COSMIC (mutation ID: COSM45304), while it has not been annotated in dbSNP and ClinVar databases. Presence of *TP53* c.375+1G>A mutation induces aberrant splicing with activation of a cryptic splice donor site in codon 59 in exon 4 [29], with a consequent frameshift and formation of a premature stop codon, as demonstrated by the functional analysis FASAY, which uses mRNA as starting material [29,30]. A germline single nucleotide polymorphism (SNP) in exon 4, the *TP53* c.215C>G, was identified in the blood and also in both HGSOC samples. This SNP codifies for a nonsynonymous/missense amino-acid (AA) change from proline (Pro) to arginine (Arg) in codon 72 (p.Pro72Arg), and it has been annotated in COSMIC (COSM250061), dbSNP (rs1042522) and ClinVar (Variation ID 12351) databases. Minor allele frequency (MAF) for SNP rs1042522 is 28.5% (G) in Eur (european) population in Ensemble database (http://grch37.ensembl.org/Homo_sapiens/Info/Index) and 34.0% (G) in ExAC Browser (http://exac.broadinstitute.org/). MAFs for *TP53* c.375+1G>A are unreported since this mutation has been described only at the somatic level. Presence of genetic polymorphism in codon 72 leads to the alteration of protein structure and decreases apoptosis. In particular, Arg at position 72 was shown to enhance p53 related apoptotic potential, rather than Pro at the same position [31,32,33,34,35]. SNP rs1042522 has been associated with a possible impact in drug response for clinical significance according to ClinVar. In the tumor samples collected at D-LPS and IDS, the SNVs identified in *TP53* are within 160 nucleotides of genomic interval from each other and are both C: G > T: A transitions. VAF for mutation *TP53* c.375+1G>A in pre-NACT chemo-naïve tumor sample was 50.13%, while in post-NACT tumor sample at IDS was 94.33%. VAF for polymorphism *TP53* c.215C>G was 27.71% and 4.21%, in pre-NACT and in post-NACT tumor samples respectively, while in blood samples at the germline level was 57.3% (Figure 2).

NGS results configured that the double SNVs identified, one in exon 4 (*TP53* c.215C>G) and one in the flanking intronic region (*TP53* c.375+1G>A) of the same exon, were in *trans* configuration [33] in the HGSOC analyzed.

### 3.3. Validation of NGS Results by Sanger Sequencing

**Figure 3:**
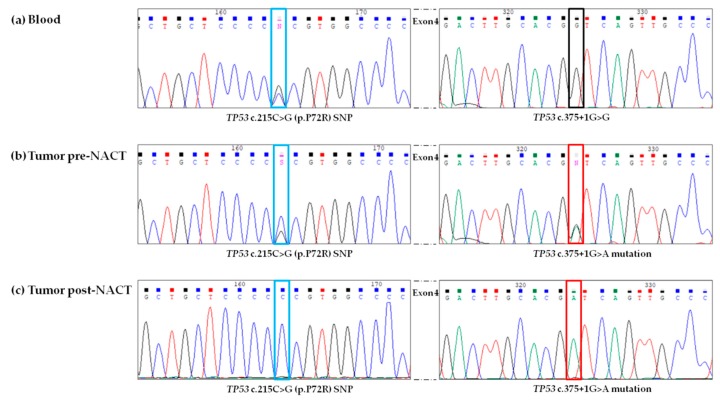
Confirmatory Sanger electropherograms of *TP53* variants identified by NGS in a patient with HGSOC, in tumor samples collected at D-LPS (pre-NACT) and at IDS (post-NACT), and in reference (germline) blood sample. (**a**) Sequencing result in the reference blood sample showing polymorphism *TP53* c.215C>G in heterozygosis (blue box) and the WT “*G*” allele at the *TP53* position c.375+1G>G (black box); (**b**) Sequencing result in the tumor sample collected at the D-LPS (pre-NACT) of the polymorphism *TP53* c.215C>G (increased peak for WT “*C*” allele, blue box) and mutation *TP53* c.375+1G>A (heterozygosis, red box), in *trans* configuration; (**c**) Sequencing result in the tumor sample collected at the IDS (post-NACT) of the polymorphism *TP53* c.215C>G indicating homozygosis of the WT “*C*” allele or loss of the Alt “*G*” allele (blue box) and mutation *TP53* c.375+1G>A indicating homozygosis of the Alt “*A*” mutated allele or loss of the WT “*G*” allele (red box), in *trans* configuration. HGSOC: high-grade serous ovarian cancer; D-LPS: diagnostic laparoscopy; IDS: interval debulking surgery; NACT: neo-adjuvant chemotherapy; SNP: single nucleotide polymorphism; NGS: next-generation sequencing; WT: wild-type.

To confirm the particular *trans* configuration of double SNVs identified in *TP53*, standard Sanger sequencing was performed using the identical genomic DNA isolated from the tumor samples and blood. Sanger sequencing confirmed NGS results and the *trans* configuration of double SNVs identified in *TP53* (Figure 3).

Further, peaks visualized in the chromatograms from Sanger sequencing were concordant with VAFs obtained with NGS approach. However, the standard sequencing approach could not discriminate allelic imbalance from a homozygous nucleotide change. In the reference (germline) blood sample, the presence of *TP53* c.215C>G polymorphism in heterozygosis and absence of the mutation *TP53* c.375+1G>A was evidenced (Figure 3a). In the pre-NACT chemo-naïve sample, the intronic alteration flanking exon 4 in heterozygous state was confirmed, as well the presence of polymorphism *TP53* c.215C>G with a diminished peak related to the “*G*” variant allele (Figure 3b). In post-NACT tumor sample, both SNVs identified showed homozygosis of Alt/mutant “*A*” allele (or suspected loss of the of the WT “*G*”) and of the WT “*C*” allele (or suspected loss of the Alt/mutant “*G*” allele), for the *TP53* c.375+1G>A and *TP53* c.215C>G variants, respectively (Figure 3c).

### 3.4. Somatic LOH

**Figure 4:**
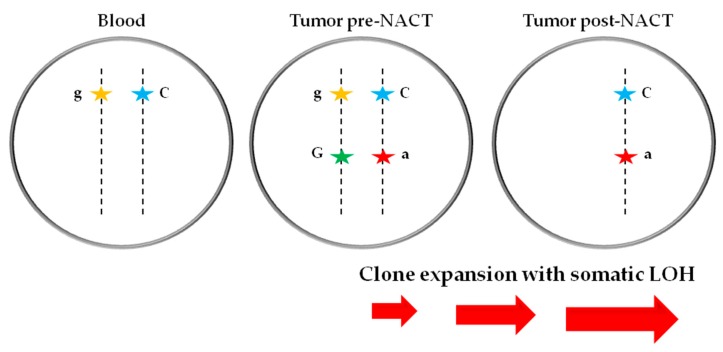
Representative model of double *TP53* SNVs in *trans* configuration identified in the pre-NACT/chemo-naïve HGSOC sample at D-LPS, with clone expansion and somatic LOH in the post-NACT tumor sample collected at IDS. In normal cells (Blood) at the germline level, only the *TP53* SNP c.215C>G was detected; in the chemo-naïve/untreated (Tumor pre-NACT) sample, the splice mutation *TP53* c.375+1G>A was present with the minor Alt allele in *trans* to the WT allele of polymorphism *TP53* c.215C>G; in the post-therapy/treated (Tumor post-NACT) sample, the complete clone expansion with somatic LOH was observed with loss of the minor Alt allele for *TP53* c.215C>G SNP and loss of the WT allele for *TP53* c.375+1G>A mutation. Alt allele of the mutation *TP53* c.375+1G>A is represented in red, the reference WT in green; Alt allele of the polymorphism *TP53* c.215C>G is colored in orange, the reference WT allele in blue; the minor Alt alleles are in lower-case letters, the WT alleles are in upper-case letters. VAF: variant allele frequency; NACT: neo-adjuvant chemotherapy; HGSOC: high-grade serous ovarian cancer; D-LPS: diagnostic laparoscopy; IDS: interval debulking surgery; Alt: alternative; WT: wild-type; LOH: loss of heterozygosity.

SNVs may be noninformative homozygous for either a reference or a variant SNV, or heterozygous and informative. In tumor cells, either the reference or variant heterozygous SNV may be entirely lost or amplified. To determine LOH, the difference and the absolute values between the tumor samples VAF and the matched normal (blood) sample VAF of the only one germline heterozygous and informative SNV identified (*TP53* c.215C>G), were calculated. The absolute differences between VAFs detected for *TP53* c.215C>G SNP in pre-NACT tumor sample, post-NACT tumor sample and blood were 29.6 and 53.1, respectively (*p* < 0.0001, for both). The germline heterozygous SNP showed significant changes in VAF, indicating allelic imbalance in the HGSOC after completion of NACT, as well in the primary untreated/chemo-naïve tumor. However, in the pre-NACT/chemo-naïve tumor sample, the somatic SNV was in heterozygosis (VAF = 50.13%) as evidenced also by the less sensitive Sanger sequencing (Figure 3b), indicating that either the sample was comprised of tumor and normal cells or clonal heterogeneity was present in the untreated tumor. SNVs identified in *trans* configuration were strongly affected by somatic LOH in the post-NACT tumor sample at IDS, with clone expansion resulting in the loss of the reference allele for *TP53* c.375+1G>A mutation and loss of the Alt/variant allele of *TP53* c.215C>G SNP (Figure 4).

### 3.5. Immunohistochemical Evaluation of p53 Expression in HGSOC Tumor Samples

**Figure 5:**
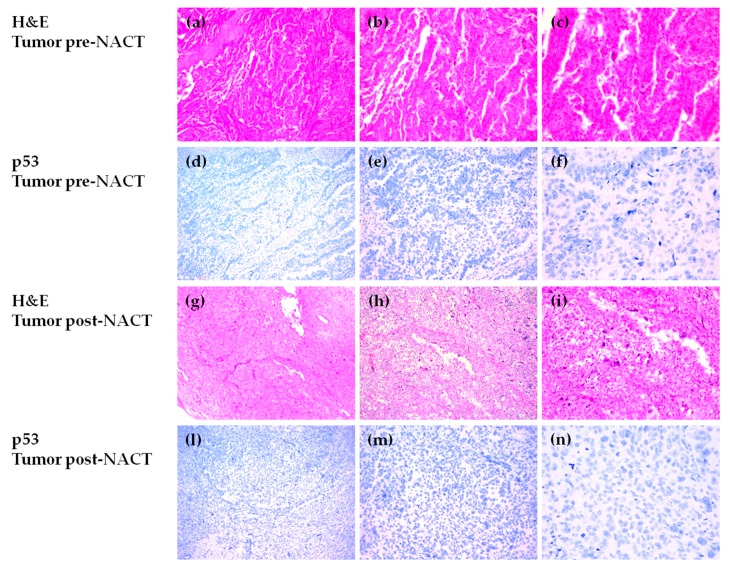
Hematoxilin and eosin (H&E, 5×, 10× and 20× magnification) of HGSOC sections collected at D-LPS (pre-NACT) and at IDS (post-NACT) and immunohistochemical staining for p53 (10×, 20× and 40× magnification). (**a**–**c**) H&E of bouin-fixed, paraffin-embedded tumor tissue collected at D-LPS showing the serous ovarian carcinoma architecture of HGSOC case with somatic mutation in *TP53* (**a**: 5×; **b**: 10×; **c**: 20×); (**d**–**f**) Absent (-) nuclear p53 expression on tumor tissue collected at D-LPS of HGSOC case with somatic mutation in *TP53* (**d**: 10×; **e**: 20×; **f**: 40×); (**g**–**i**) H&E of formalin-fixed, paraffin-embedded tumor tissue collected at IDS showing the serous ovarian carcinoma architecture of HGSOC case with somatic mutation in *TP53* (**g**: 5×; **h**: 10×; **i**: 20×); (**l**–**n**) Absent (-) nuclear p53 expression on tumor tissue collected at IDS of HGSOC case with somatic mutation in *TP53* (**l**: 10×; **m**: 20×; **n**: 40×). No cytoplasmic staining was observed. NACT: neo-adjuvant chemotherapy; HGSOC: high-grade serous ovarian cancer; D-LPS: diagnostic laparoscopy; IDS: interval debulking surgery.

HGSOC showed papillary, glandular, and solid architecture with psammoma bodies in D-LPS/pre-NACT (Figure 5a–c) and micropapillary morphology in IDS/post-NACT (Figure 5g–i) tumor tissue samples.

Severe nuclear atypia (multinucleated tumor giant cells) was observed in both pre- and post-NACT samples, in particular in chemo-naïve sample (Figure 5c). Immunohistochemical staining of p53 was completely negative (-) in tumor cell nuclei and cytoplasm, in both D-LPS/pre-NACT (Figure 5d–f) and IDS/post-NACT (Figure 5l–n) tissue samples. In the primary chemo-naïve tumor, BRCA1, p16, WT1, and Ki-67 markers were also evaluated by immunohistochemistry analysis.

**Figure 6:**
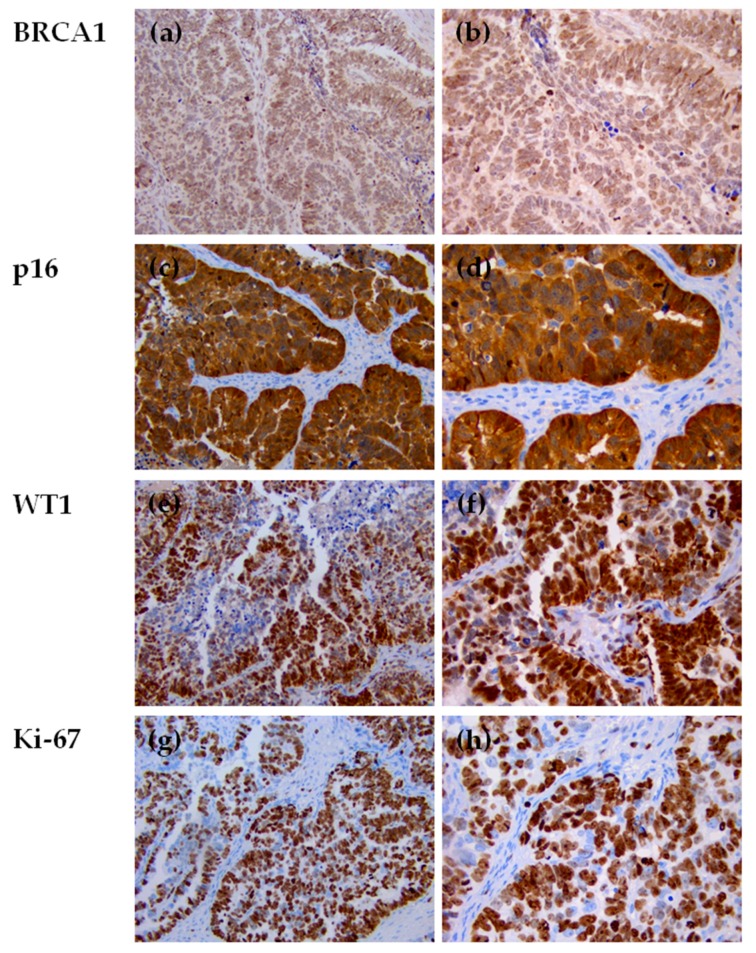
Immunohistochemical staining for BRCA1, p16, WT1, and Ki-67 of HGSOC sections collected at D-LPS from a patient diagnosed of HGSOC, before NACT (20× and 40× magnification). (**a**,**b**) Positive nuclear staining for BRCA1 observed in ~95% of tumor cells (**a**: 20×, **b**: 40×); (**c**,**d**) Diffuse nuclear and cytoplasmic staining of p16 exhibited by ~95% of tumor cells (**c**: 20×, **d**: 40×); (**e**,**f**) Nuclear and cytoplasmic staining for WT1 observed in ~70% of tumor cells (**e**: 20×, **f**: 40×);(**g**,**h**) Diffuse nuclear staining of Ki-67 observed in ~95% of tumor cells (**g**: 20×, **h**: 40×). NACT: neo-adjuvant chemotherapy; HGSOC: high-grade serous ovarian cancer; D-LPS: diagnostic laparoscopy; BRCA1: BRCA1 DNA repair associated; WT1: Wilms tumor 1.

BRCA1 expression was positive in about 95% of tumor cells with a weak (+) nuclear staining intensity (Figure 6a,b).

Diffuse nuclear and cytoplasmic staining was observed for p16 (i.e., cyclin dependent kinase inhibitor 2A (CDKN2A) protein) in ~95% of tumor cells with ~20% of cells with weak (+), ~55% with moderate (++) and ~20% with high (+++) intensity (Figure 6c,d). Nuclear and cytoplasmic staining for WT1 protein was exhibited in ~70% of tumor cells: weak (+) intensity was observed in the cytoplasm while nuclear staining showed ~20% of cells with weak (+), ~20% with moderate (++) and ~30% with high (+++) intensity (Figure 6e,f). The percentage of Ki-67 positive tumor nuclei (Ki-67 proliferation index) [34] was ~95% with ~20% of cells with weak (+), ~40% with moderate (++) and ~35% with high (++) staining intensity (Figure 6g,h).

## 4. Discussion

In advanced ovarian cancer, the benefit from NACT based on platinum and paclitaxel followed by IDS, is still a matter of debate. The possibility that NACT may induce chemoresistance before and after IDS has been poorly investigated [6,8,15,16]. By means of targeted NGS, matched pre-and post-neoadjuvant platinum/paclitaxel-based chemotherapy tumor samples, as well the normal/blood reference sample, from a patient with stage IV HGSOC treated with NACT-IDS, were analyzed. Expansion of the *TP53* c.375+1G>A driver mutant clone with somatic LOH and loss of the 72Arg allele of p53 after NACT were observed, suggesting a possible mechanism of chemoresistance in HGSOC. The patient had a very aggressive clinical course of ovarian disease, which underlined the aggressive tumor phenotype. Immunohistochemical analysis indicated that tumor cells were highly proliferative (Ki-67) and exhibited a typical phenotype of HGSOC with the expression of p16 and WT1 markers. Expression of the Ki-67 marker is strongly associated with cell proliferation and is commonly used in routine diagnostic pathology [36], also to distinguish HGSOC from low-grade serous ovarian cancer (LGSOC) [37]. WT1 is not expressed in the epithelium of healthy fallopian tubes, which is assumed to be the site of origin of most HGSOC [38], and it is a recognized marker of serous differentiation, useful to distinguish serous tumors (both high-grade from low-grade) from other tumor types [39]. Diffuse p16 staining supports a diagnosis of HGSOC, especially when p53 expression is uncertain [40]. The prognostic value of WT1 expression marker has been also investigated in HGSOC, but with opposite effects [38,41]. The analyzed chemo-naïve tumor showed WT1 positive, absent p53 and diffuse p16 immunostaining, which is concordant with HGSOC phenotype; conversely, LGSOC are characterized by positive expression of WT1 and by WT pattern for p53 and patchy for p16 [39]. The patient was refractory to treatment after four cycles of platinum, and despite CA-125 normalization levels, she had a poor pathological response at the end of the following six cycles with the addition of paclitaxel. No increase in the tumor mutational or neoantigen load was found when comparing tumor samples prospectively collected before and after NACT. This result is in agreement with that reported for other studies in urothelial [42] and in bladder [43] primary carcinomas, in which matched pre- and post-neoadjuvant cisplatin-based chemotherapy tumor samples were analyzed by NGS. The somatic mutation identified in *TP53* was unique in both matched pre-and post-therapy tumor samples, similarly to recent previous research in HGSOC [22,44]. Direct induction of *TP53* mutations by DNA-damaging chemotherapy with alkylating agents is a possible recognized event [45]. The first relevant finding in this study is that the somatic mutation identified in *TP53* was not a consequence of DNA damaging drugs because it arose before the start of chemotherapy treatment. This result is also suggestive that the driver splice mutation detected conferred a selective advantage and in vivo chemotherapy resistance, being the only one somatic variant identified in post-therapy sample, according to the 26 cancer-genes panel used. Indeed, the selection of pre-existing mutated clones by chemotherapy was reported as the predominant mechanism of *TP53* mutation expansion [46,47]. However, the association of *TP53* mutations with chemoresistance in cancer [48] and in HGSOC [23,49] is still controversial. The splice variant *TP53* c.375+1G>A, affecting a consensus SD site, is an infrequent somatic mutation (among those reported for *TP53*) already described in sporadic breast cancer non-carriers in *BRCA1* mutations [50], in situ skin cancer lesions [51], bladder cancer [52], esophageal squamous cell carcinoma [29,53], ovarian cancer [54], HGSOC [55], and in other type of malignancies as currently reported in COSMIC database. Other somatic mutations affecting the consensus (*TP53* c.375+1G>T, *TP53* c.375+2T>A) and non-consensus (*TP53* c.375+5G>A) SD site in exon 4 flanking region of *TP53* have been already reported in different malignancies, including HGSOC, and they were associated with similar altered splicing of *TP53* c.375+1G>A, with additional aberrations (i.e., insertion of whole intron 4 and frameshift, insertion of 109 nucleotides of intron 4 and frameshift) [30]. We also evaluated, with the same targeted NGS panel used in this study, the entire coding region of *TP53* and the related intronic boundaries in 79 chemo-naïve advanced (III-IV stage, tumor grade G2-3) ovarian tumors, finding splice site mutations only in the sub-group of HGSOCs, in six patients (11.5%) [23]. Intriguingly, three out of six patients had somatic splice mutations affecting directly (*TP53* c.375+1G>T and *TP53* c.375+2T>A), and non-directly (*TP53* c.375+5G>A), the SD site in exon 4 flanking region of *TP53* [23]. Moreover, all six patients were also carriers of the polymorphism *TP53* p.Pro72Arg and the one mutated for *TP53* c.375+5G>A had also a concurrent mutation in *KRAS* (*KRAS* p.G12C), contributing to define a poor prognostic signature in patients with HGSOC [23]. These patients with somatic splice mutations in intron 4 of *TP53* were also characterized by a poor prognosis (median PFI < 4 months, median TTR < 12 months, median OS < 27 months).

The amount of mutations in *TP53* implicated in splice junctions that interfere with correct protein translation, by introducing frameshift or aberrant spicing, has been probably underestimated in many studies, due to the restriction of genetic analyses in coding regions and in the DNA binding domain (DBD) of p53. Many rare genomic alterations, such as those affecting the splice site regions, have been unveiled by the application of NGS, thus expanding the potential repertoire for the use of target therapies and personalized therapy [24]. Aberrant splicing events are a common phenomenon in tumorigenesis described in each of the accepted hallmarks of cancer, particularly in apoptosis and metastasis formation, even if, their biological functions are still not completely clarified [56]. Splice site mutations when categorized as disruptive or predicting a non-functional p53 protein together with nonsense and frameshift variants [57] have shown a poor prognostic value in squamous head and neck cancer [58] and in a large cohort of patients with breast cancer [59]. Moreover, disruptive *TP53* mutations showed significant radioresistance with a failure of senescence induction, compared to nondisruptive/missense mutations [58]. The downstream effects of splice mutations on target gene expression and pathway activation are still poorly investigated. Nonetheless, some truncated p53 isoforms associated to a p53-null state named p53 ψ, derived from splice acceptor (SA) site mutations (*TP53* c.673-2A>T/C/G; *TP53* c.673-1G>T/C/A), are capable of reprogramming cells inducing the acquisition of pro-metastatic features [60,61]. A recent study evaluated the amount of *TP53* splice mutations through the sequencing of exons 2-11 and ten intronic flanking nucleotides for each exon, in a series of 401 primary non-metastatic colorectal cancers (CRCs): the number of these type of mutations detected was three-fold higher than reported in major databases [62]. Furthermore, splice mutations were found to be significantly associated with worse relapse-free survival, defining a high-risk group for stage II CRC [62].

The *TP53* c.375+1G>A mutation affects directly p53 splicing [29] producing a short truncated protein without most of known coding sequence, including the functional DBD and the oligomerization domain (OD), which contains also the localization and exportation signals for nuclear importation and exportation of p53, respectively [25]. Therefore, unsurprisingly, expression of p53 in both pre- and post-NACT tumor samples, that showed completely negative immunostaining, indicating abnormal p53 expression [39], and loss of the p53-WT functions, was not observed. Notably, negative p53 tumor staining was concordantly reported in two patients with primary/chemo-naïve HGSOC, one with the same somatic mutation *TP53* c.375+1G>A, and the other mutated for *TP53* c.375+2T>G, affecting the analogous SD site [55].

Codon 72 of p53 human protein is localized in the proline-rich domain (residues 58–101), a hydrophobic region characterized by five copies of sequence PXXP, whereas P is Pro and X any AA residue [63]. This motif forms a left-handed polyPro type II helix which creates a binding site for src homology-3 (SH3) domain [64]. The relevance of the PXXP sequence is supported by the fact that this motif has been found in all the proteins known to bind directly to SH3 domains [65]. The PXXP motif, due to its conservation in different species, is considered a discrete signal module playing a critical role in the transmission of antiproliferative signals [65]. Moreover, p53 mutants lacking 62-91 residues demonstrated that the proline-rich domain is required to induce apoptosis and cell death in presence of compounds used in cancer treatment causing DNA damage, which sensitize cells to p53 dependent apoptosis [63]. When cells without the five PXXP domains were treated with different chemotherapeutic drugs (camptothecin, colchicines, daunorubicin, etoposide, 5-fluorouracil), they failed to undergo apoptosis [63]. Genetic polymorphism in codon 72 with AA exchange from Pro to Arg, leads to the loss of one of the five PXXP repeats with alteration of p53 protein structure and p53-dependent apoptosis induction [31,32,66]. In particular, Arg at position 72 was shown to enhance the apoptotic potential more than Pro at the same position, leading mutated cells with 72Arg allele, to acquire an increased sensitivity with a more vigorous apoptotic response after chemotherapy treatment [35,66,67], partially through targeting p53 to the mitochondria [31]. Presence of the 72Pro allele has been associated with poor survival and prognosis in different tumors [34,35,54,68]. In particular, our group reported that patients with high-grade osteosarcoma were associated to significant reduced survival when homozygous for 72Pro allele [35].

In the HGSOC here presented, if the identified variants, somatic and germline, were in *cis* configuration in the tumor, the driver *TP53* splice mutation could have been coupled with the “pro-apoptotic” 72Arg allele, that may sustains the damaging effects of chemotherapy favoring tumor cell death. The particular *trans* configuration between the germline SNP and the somatic driver mutation suggests that it is critical for this tumor because it provides a selective advantage to cancer cells regardless of germline background. Somatic or germline variants have been mostly independently analyzed in cancer genome studies. Increasing evidences are focusing on the intricate linkage between germline and somatic variants which consequence is, from a molecular and phenotypic standpoint, the complex cancer disease, influenced by both inherited variants in germline DNA and somatic alterations acquired during formation of the tumor [69]. Germline variants are estimated, through genome-wide association studies [70], to contribute for a 20% to cancer development and have been reported to influence gene expression in tumors [69,71]. In ovarian cancer, a recent study integrated data from rare germline and somatic mutations identifying novel genes and variants of potential impact in ovarian cancer susceptibility [72]. Inactivation of tumor-suppressor genes and activation of oncogenes which finally lead to uncontrolled cell growth and metastatic spread, may depend on genetic alterations, including LOH and copy number variations (CNVs) [73]. In ovarian cancer, LOH of the WT allele is recognized as the tumor-initiating second hit in most of patients with a pathogenic germline mutation in *BRCA1* or *BRCA2* genes [72,74,75]. BRCA1 immunostaining has a negative predictive value of 100% for germline mutations in *BRCA1* (intact/positive staining corresponds to lack of *BRCA1* mutations) [76]. Furthermore, 94% of HGSOCs without homologous recombination (HR) alterations in DNA repair pathway (most commonly in *BRCA1/2* genes) evaluated by NGS, exhibited positive BRCA1 expression [77]. We observed positive immunostaining for BRCA1 protein in the pre-NACT tumor sample from the HGSOC analyzed, suggesting the lack of germline (and probably somatic) *BRCA1* alterations. This result is concordant with the unreported family history of ovarian and breast cancer and with unfavorable prognosis of the patient since carriers of either germline or somatic *BRCA1* mutations are associated with improved survival compared to patients with non-*BRCA* mutations [78]. Furthermore, the somatic mutations in upstream HR modulators, such as in *PTEN* (included in the 26-cancer genes panel), that may induce a “BRCAness” phenotype [23], were not detected.

The cooperation between germline and somatic variants that often requires a close interaction to develop cancer is best exemplified by the “two-hit hypothesis” [79], in which a tumor suppressor gene is inactivated by the combination of an initial germline mutation of one allele, followed by the somatic inactivation of the other [80]. LOH, whereby the WT allele for a two-hit tumor suppressor is eliminated, has been implicated in many cancers, including ovarian [72]. Nevertheless, somatic LOH may result in the loss of either the variant or the reference allele in the tumor, respectively [26]. LOH analysis through the NGS approach can easily uncover heterozygous germline variants that are under potential selection in the tumor, one of the key indications is the significant increased or decreased VAF in the tumor sample [80]. Through the application of NGS technology, the comparison of SNP allele frequencies from paired tumor and normal DNA to evaluate whether heterozygous loci in the blood/normal reference DNA sample are turned into homozygous loci in the tumor DNA sample, represents a simple method to detect LOH [26,27,72,81]. To investigate the possibility that somatic LOH could increase functional impact of the germline heterozygous SNV *TP53* c.215C>G (p.Pro72Arg) detected in the tumor samples, we took advantage of the quantitative nature of NGS technique, which has a higher sensitivity than microsallite-based LOH and multiplex ligation-dependent probe amplification [82]. Although in the chemo-naïve HGSOC the VAF decrease for the informative germline SNV was significant and suggestive of LOH, heterozygosity for the somatic mutation *TP53* c.375+1G>A was clearly detected in the pre-NACT tumor sample by NGS and also by the less sensitive Sanger sequencing method. The pre-NACT tumor sample was probably not completely free of normal tissue and may have exhibited subclonal heterogeneity [27]. Notably, VAF > 70%, related to only somatic mutations identified, was used to assess somatic LOH in 72 cases of primary HGSOC [55]: VAF related to the *TP53* c.375+1G>A somatic mutation overcome this threshold only in the post-NACT tumor sample. Overall, LOH was present in the post-NACT tumor sample with elimination, under the selective pressure of NACT, of the treatment-sensitive 72Arg allele and concomitant expansion of the variant/mutant allele of a driver splice site mutation in *TP53* intron 4, in *trans* configuration with respect to the *TP53* p.Pro72Arg SNP.

When arising in patients who are *TP53* p.Pro72Arg germline heterozygotes, the 72Pro allele has been reported to be preferentially retained in primary ovarian [54] and breast [34] cancers, through LOH. Of note, in patients with chemo-naïve ovarian carcinoma, women homozygous for the 72Pro allele had a significant shorter survival compared to those who were carriers for an Arg allele: eighty-four percent of *TP53* p.Pro72Arg germline heterozygotes showed somatic LOH (56% retained 72Pro allele and 44% retained 72Arg allele), but among the *TP53* mutated tumors who exhibited LOH there was no preference in loss of either Pro or Arg allele [54]. Hence, this study reports for the first time, in one patient with HGSOC, a selective retention of the germline 72Pro allele after NACT treatment.

Clonal evolution has been studied in different malignancies [21,42,46,47,83,84,85]. Selective pressure induced by chemotherapy is exerted through the selection of mutations that confer proliferative or chemotherapy resistance advantages; thus, treatment-sensitive clones are eliminated, whereas refractory/resistant clones tend to become dominant [86,87]. In urothelial carcinoma, characterized by resistance to platinum-based treatment and rapid tumor progression, selective pressure exerted by chemotherapy has been investigated by analyzing (by NGS) matched sets of primary advanced tumors collected before and after chemo-treatment, demonstrating clonal enrichment in post-chemotherapy tumor samples [42,83].

One important strength of this study is that, to our knowledge, it is the only investigation on clonal evolution of chemotherapy-refractory/resistant HGSOC after NACT, with relevance in translational medicine. The limitations of our study are due to the findings in only one case that should be explored and confirmed in other patients with HGSOC that undergo NACT based on platinum/paclitaxel therapy. Another limit is represented by the unexplored mutational profile in the metastatic sites, such as in pleural effusion or in abdominal ascites from this patient, and also from relapse/solid biopsies. Molecular profiling of different matched samples from the same patient may shed light on the comprehension of the mutagenic pressure from the primary untreated tumor, to the advanced and chemotherapy treated one, at different anatomic sites. Another possible application which could improve this type of study, is represented also by the characterization of the mutational profile in the liquid biopsies through analysis of circulating free DNA. Thus, further investigations are needed to provide new insights on the evolution of HGSOC under the neo-adjuvant interventions.

## 5. Conclusions

In summary, this study reports, in a patient with stage IV HGSOC characterized by poor response to treatment and prognosis, that the *TP53* c.375+1G>A somatic mutation affecting a consensus SD site in intron 4 flanking region was an early mutational event, and it was shared in the tumor before and after NACT. SNV identified in the primary chemo-naïve HGSOC preceded clonal evolution exhibited in the tumor sample exposed to NACT, after the selective pressure of platinum/paclitaxel treatment. Expansion of the founder clone with the *TP53* driver mutation through LOH was coupled with the retention of the 72Pro allele and the loss of the “pro-apoptotic” 72Arg allele, codified by the germline heterozygous SNP *TP53* c.215C>G (p.Pro72Arg) in exon 4. LOH involving the 72Arg allele and the WT allele of the driver somatic SD mutation of *TP53* in the resistant treated tumor, suggests that this event may play a role in chemoresistance. This dominant mutation in the resistant tumor should be considered as a clearly negative factor that may impact the patient’s outcome. The assessment of the SNP *TP53* c.215C>G should be also considered to verify the *trans* or *cis* pattern with respect to *TP53* c.375+1G>A mutation and also over other splice mutations affecting directly and non-directly the SD site flanking exon 4 in *TP53*. Patients with *trans* configuration of these SNVs in the tumor are at high risk of mutation selection by NACT based on platinum/paclitaxel combination therapy. Our study highlights the interplay between germline and somatic variants under the selective pressure of NACT and may have a relevant impact in translational medicine.

## Figures and Tables

**Table 1:** *TP53* SNVs identified by NGS in a patient with HGSOC treated with NACT.

Blood	Tumor	Tumor	*TP53* Gene	Genomic	cDNA	Ref_	Alt	Variant	AA	Predicted
(Germline)	Pre-NACT	Post-NACT	Region	Coordinate *	Change	seq	_seq	Type	Change	Protein Product
No	Yes	Yes	IVS4	17:7,579,311	c.375+1G>A	G	A	SD	p.Gly59Valfs*23	Truncated
Yes	Yes	Yes	Exon 4	17:7,579,472	c.215C>G	C	G	Missense	p.Pro72Arg	Full-lenght

* Reference build is GRC37/h19. SNV: single nucleotide variant; NGS: next-generation sequencing; HGSOC: high-grade serous ovarian cancer; VAF: variant allele frequency; Ref: reference allele; Alt: alternative allele; AA: amino acid; SD: splice donor; NACT: neo-adjuvant chemotherapy; IVS4: intervening sequence 4, i.e., intron sequence 4; Gly: glycine; Val: valine; fs: frameshift; Pro: proline; Arg: arginine.
